# Chinese visceral adiposity index outperforms insulin resistance indices in predicting cardiovascular disease risk: a 12-year prospective cohort study

**DOI:** 10.3389/fnut.2026.1795750

**Published:** 2026-05-05

**Authors:** Weiyan Tu, Rongdi Xu, Na Luo, Meijing Wu, Yong Zhou, Jing Ning

**Affiliations:** 1 Shanghai Key Laboratory of Maternal-Fetal Medicine, Shanghai Institute of Maternal-Fetal Medicine and Gynecologic Oncology, Shanghai First Maternity and Infant Hospital, School of Medicine, Tongji University, Shanghai, China; 2 Department of Obstetrics, International Peace Maternity and Child Health Hospital, School of Medicine, Shanghai Jiao Tong University, Shanghai, China; 3 Hainan Branch, Shanghai Children’s Medical Center, School of Medicine, Shanghai Jiao Tong University, Sanya, China; 4 Clinical Research Institute, Shanghai General Hospital, Shanghai Jiao Tong University School of Medicine, Shanghai, China

**Keywords:** cardiovascular disease, Chinese visceral adiposity index, cohort study, insulin resistance, risk prediction

## Abstract

**Aims:**

This study investigated and compared the abilities of seven insulin resistance (IR) indices, including the triglyceride-glucose index (TyG), TyG-waist circumference (TyG-WC), TyG-waist-to-hip ratio (TyG-WHtR), lipid accumulation product (LAP), visceral adiposity index (VAI), Chinese visceral adiposity index (CVAI), and metabolic score for insulin resistance (METS-IR) to predict cardiovascular disease (CVD) among participants without a history of CVD.

**Methods:**

The population was recruited from the prospective Asymptomatic Polyvascular Abnormalities Community (APAC) Study in Hebei. This prospective study included 5,194 individuals without history of CVD who were stratified into CVD group and non-CVD group. The seven IR indices (TyG, TyG-WC, TyG-WHtR, LAP, VAI, CVAI, and METS-IR) were calculated. The primary endpoint was CVD.

**Results:**

Over a 12-year follow-up period, 373(7.2%) CVD were documented. The seven IR indices were strongly positively associated with CVD risk. Compare to other IR indices, CVAI was the strongest predictor for CVD (HR:1.84 95% CI: 1.45–2.35; *p* < 0.001) and showed the highest diagnostic value for CVD (AUC: 0.654 95% CI: 0.640–0.666; *p* < 0.001). Adding CVAI to the based model exhibited the maximum increased on incremental risk stratification for CVD (NRI: 0.355; IDI: 0.010; all *p* < 0.001). Moreover, there was a non-linear association between CVAI and CVD, with a threshold point of 96.21. The association between CVAI and CVD risk was more prominent among individuals aged<65 years, female, and non-hypertension (all P interaction<0.001).

**Conclusion:**

Compared to the other six tested IR indices, CVAI might be the most valuable index to predict and stratify CVD risk, with enhanced prediction in women under 65 years of age. This suggests that strategies aimed at improving risk stratification should incorporate CVAI into risk algorithms.

## 1 Introduction

Cardiovascular disease (CVD) poses a major global public health burden, with persistently high morbidity and mortality, particularly among the elderly population (1). A substantial burden of ischemic heart disease, ischemic stroke, and peripheral artery disease persists worldwide. Moreover, significant inequalities in cardiovascular care quality exist across regions, socioeconomic levels, and genders, highlighting the urgent need for optimized risk assessment and management strategies (1). In 2019, a total of 17.9 million people lives were lost to CVD, and by 2030 it is projected to increase by 22.2 million (2). Despite the widespread adoption of primary and secondary prevention strategies for CVD, there has been limited improvement in the prevalence of CVD. Thus, it is necessary to identify the effects of risk factors on CVD in order to implement risk stratification and precise management.

Insulin resistance (IR), a key component of metabolic syndrome, which includes hypertension, obesity, diabetes (3), and hyperlipidemia (4), is associated with an increased risk of CVD (5, 6). The hyperinsulinaemic-euglycaemic clamp (HIEG) and the homeostasis model assessment of insulin resistance (HOMA-IR), considered the gold standard for diagnosing IR, are operationally complex, time-consuming, and costly, making them unsuitable for clinical detection of IR. Therefore, considering the characteristics mentioned above, seven IR indices including triglyceride-glucose index (TyG), TyG-WC, TyG-WHtR, visceral adiposity index (VAI), Chinese visceral adiposity index (CVAI), lipid accumulation product (LAP), and metabolic score for insulin resistance (METS-IR) are performed by common laboratory and anthropometric parameters, which have been strongly associated with HIEG clamp (7–9). Numerous studies have suggested that these IR are associated with the risk of CVD and adverse prognosis (10–14). However, it remains unclear which IR surrogate has the strongest predictive and stratification ability for CVD. In contrast, other surrogates, such as the TG/HDL-C ratio, are single biochemical marker that only reflects lipid metabolism and lacks the comprehensive assessment of adiposity and metabolism provided by the seven IR indices. Its predictive performance is inferior to that of the TyG index series in some studies (15). METS-VF has relatively limited supporting evidence in the Chinese population and requires more complex measurement parameters, making it less suitable for the present study cohort. Therefore, this study aims to systematically explore the predictive and stratification abilities of different IR indices for CVD risk among the general population without a history of CVD.

## 2 Methods

### 2.1 Study population

The participants were older than 40 years and resided in Tangshan, Hebei, Northern China. The population was recruited from the prospective APAC Study, which aims to research the incidence and correlations of asymptomatic polyvascular abnormalities (APA) among the general public. The APAC study commenced in June 2010, including 5,440 eligible participants who had no history of stroke, transient ischemic attack, or coronary disease at baseline, as assessed by a validated questionnaire (16). The exclusion criteria were as follows: (1) participants who had malignant tumor (*n* = 54); (2) those with missing data on fasting plasma glucose (FPG), triglyceride (TG), high-density lipoprotein-cholesterol (HDL-C) (*n* = 110); (3) those with lacking waist circumference (WC) and height (*n* = 82). Finally, 5,194 individuals were enrolled in the current study. The individuals were stratified into CVD group (*n* = 373) and non-CVD (*n* = 4,812) (Supplementary Figure 1).

### 2.2 Data collection and definition

Researchers collected baseline data from participants by using standardized questionnaires. These included demographic information, history of disease, lifestyles, and blood biochemical measurements. The clinical characteristics and biochemical indicators were assessed at Kailuan General Hospital. The detailed protocol and assessment standard were described in a previous study. The Body Mass Index (BMI) was calculated as weight (kg)/height (m^2^). The formulas for seven insulin resistance indices (TyG, TyG-WC, TyG-WHtR, LAP, VAI, CVAI, and METS-IR) were provided in Supplementary Table 1.

### 2.3 Follow-up and outcomes

All participants were followed through face-to-face interviews at each 2-year routine medical examination until death, or until December 31, 2022. When face-to-face meetings were not feasible, participants were followed up for 2 years using available medical insurance records, and with follow-up extended as additional clinical data were available. The primary outcome was CVD, defined as a composite of stroke and myocardial infarction (MI). Myocardial infarction (MI) and stroke were combined to identify CVD in this study using data from discharge records and biennial questionnaires. Myocardial infarction and stroke were identified using ICD-10 codes I21 and I63, I60, or I61, respectively. Medical records of likely CVD events were reviewed by three physicians who were unaware of the study’s objective.

### 2.4 Statistical analysis

Continuous variables were tested for normality using the Shapiro–Wilk test and are shown as mean ± standard deviation if normally distributed, while skewed distributed data are shown as medians with interquartile ranges. Categorical variables are presented as frequencies and percentages. For continuous variables, normally distributed data were tested for independent sample t-tests, and skewed distributed data were tested for Mann–Whitney U tests. The chi-squared test was used for categorical variables.

Kaplan–Meier survival curves were constructed to evaluate CVD according to seven IR indices (TyG index, TyG-WC, TyG-WHtR, LAP, VAI, CVAI and METS-IR). The study utilized a Cox regression model to evaluate the relationship between various IR indices and CVD. Thereafter, variables demonstrating a significant association (i.e., *p* < 0.05) with CVD were integrated into multivariate Cox regression, which was adjusted for age, sex, smoking, education level, hypertension, dyslipidemia, diabetes, creatinine, and homocysteine. All the adjusted variables were evaluated for collinearity, and no clear evidence of multicollinearity was detected (the all-variance inflation factor of the included variables was < 2) (Supplementary Figure 2). Restricted cubic spline (RCS) curve analysis was performed to identify linear or non-linear relationships between various IR indices and the risk of CVD. 5 knots were used for the RCS model, which were placed at the 5th, 25th, 50th, 75th, and 95th percentiles of various IR indices distribution. Receiver operating characteristic curves (ROC) were used to evaluate the performance of these IR indices for predicting CVD risk values. The DeLong test was conducted to compare the AUCs of IR indices. To determine the discriminatory and reclassification ability of IR indices over the base model (including age, sex, smoking, education level, hypertension, dyslipidemia, diabetes, creatinine, and homocysteine) for predicting CVD risk, integrated discrimination improvement (IDI), and net reclassification improvement (NRI) were calculated.

The threshold analysis estimated the inflection point, and further investigated the association between CVAI and the risk of CVD using a two-piecewise Cox proportional risk model. Stratified analyses according to age (< 65 years and ≥ 65 years), gender, hypertension, and diabetes were performed to assess potential modification effects. Finally, Sensitivity analyses were conducted to confirm the robustness of the findings. To assess potential bias for residual confounding by anti-diabetes and anti- dyslipidemia, we repeated the analyses separately, excluding participants with anti-diabetes or anti-dyslipidemia. To further validate the robustness of our primary findings and reduce biases from early follow-up events, a prespecified sensitivity analysis was performed by excluding all study endpoints occurring within the first 2 years. A two-sided *p* < 0.05 was considered to be statistically significant. Statistical analysis was performed using R version 4.1.2 (R Foundation for Statistical Computing, Vienna, Austria).

## 3 Results

### 3.1 Baseline characteristics

Table 1 showed the baseline characteristics of the participants. A total of 5,194 participants fulfilled the final analysis, with a mean age of 54.8 ± 11.6 years, and 59.9% of participants were male. The levels of IR indices (TyG index, TyG-WC, TyG-WHtR, LAP, VAI, CVAI, and METS-IR) were all significantly higher among individuals with CVD. Participants in the CVD group tended to be older, more proportions of males, education level, smoking, drinking, hypertension, DM, hyperlipidemia, higher levels of WC, BMI, SBP, DBP, TG, TC, FBG, creatinine, and homocysteine, while lower levels of HDL-C.

**Table 1:** Baseline characteristics of the total individuals.

Variable	Overall (*n* = 5,194)	CVD (*n* = 373)	Non-CVD (*n* = 4,821)	*p*-value
Age (y)	54.8 ± 11.6	60.5 ± 11.7	54.4 ± 11.5	<0.001
Male, *n* (%)	3,109 (59.9)	270 (72.4)	2,839 (58.9)	<0.001
Education level, *n* (%)				<0.001
Primary school	612 (11.8)	65 (17.4)	547 (11.3)	<0.001
Middle school	2,296 (44.2)	143 (38.3)	2,153 (44.7)	<0.001
High school or above	2,286 (44.0)	165 (44.2)	2,121 (44.0)	<0.001
Smoker, *n* (%)	1,662 (32.0)	133 (35.7)	1,529 (31.7)	0.116
Drinker, *n* (%)	746 (14.4)	62 (16.6)	684 (14.2)	0.197
Diabetes, *n* (%)	609 (11.7)	71 (19.0)	538 (11.2)	<0.001
Hypertension, *n* (%)	2,469 (47.5)	245 (65.7)	2,224 (46.1)	<0.001
Hyperlipidemia, *n* (%)	2,514 (48.4)	213 (57.1)	2,301 (47.7)	<0.001
Anti-dyslipidemia, *n* (%)	68(1.3)	8 (2.1)	60 (1.2)	0.273
Anti-diabetic, *n* (%)	385 (7.4)	51 (13.7)	334 (6.9)	<0.001
WC, cm	86 (80,93)	89 (83,95)	86 (79,92)	<0.001
WHtR	0.52 (0.48,0.55)	0.53 (0.50,0.57)	0.52 (0.48,0.55)	<0.001
BMI, kg/m^2^	24.75 (22.68,27.04)	25.10 (23.27,27.43)	24.69 (22.66,27.04)	0.016
SBP, mmHg	130 (120,140)	138 (125,150)	130 (119,140)	<0.001
DBP, mmHg	80 (76,90)	84 (80,90)	81 (76,90)	<0.001
TG, mmol/L	1.31 (0.94,1.93)	1.42 (0.99,2.06)	1.30 (0.93,1.92)	0.003
TC, mmol/L	4.95 (4.35,5.62)	5.03 (4.44,5.77)	4.93 (4.35,5.61)	0.021
LDL-C, mmol/L	2.60 (2.17,3.05)	2.60 (2.13,3.10)	2.60 (2.17,3.05)	0.719
HDL-C, mmol/L	1.57 (1.30,1.89)	1.52 (1.23,1.83)	1.57 (1.31,1.90)	0.003
FPG, mmol/L	5.20 (4.82,5.80)	5.34 (4.88,6.06)	5.20 (4.82,5.78)	<0.001
Creatinine, μmol/L	72 (61,84)	74 (65,85)	72 (61,84)	0.005
Homocysteine, μmol/L	13.50 (9.60,19.20)	14.66 (10.43,20.90)	13.40 (9.50,19.10)	<0.001
TyG	8.62 (8.24,9.08)	8.76 (8.42,9.26)	8.61 (8.24,9.06)	<0.001
TyG-WC	744.09 (668.39,826.50)	794.22 (723.67,889.27)	741.15 (664.07,821.33)	<0.001
TyG-WHtR	4.48 (4.07,4.91)	4.74 (4.32,5.10)	4.46 (4.05,4.89)	<0.001
LAP	30.69 (17.82,52.07)	39.16 (23.99,68.39)	30.09 (17.51,50.82)	<0.001
VAI	1.25 (0.82,2.02)	1.41 (0.94,2.30)	1.24 (0.81,1.99)	<0.001
CVAI	95.37 (66.97,123.04)	114.73 (89.82,140.44)	93.72 (65.74,120.92)	<0.001
METS-IR	31.87 (28.35,36.01)	33.55 (30.33,38.14)	31.74 (28.21,35.87)	<0.001

Data are expressed as mean ± standard deviation, median with 25th and 75th percentiles, or *n* (%). BMI body mass index, SBP systolic blood pressure, DBP diastolic blood pressure, FPG fasting plasma glucose, TC total cholesterol, TG triglyceride, HDL-C high-density lipoprotein-cholesterol, LDL-C low-density lipoprotein-cholesterol, TyG index, the triglyceride-glucose index. TyG triglyceride-glucose, VAI visceral adiposity index, CVAI Chinese visceral adiposity index, LAP lipid accumulation product, METS-IR metabolic score for insulin resistance, WC waist circumference, WHtR waist-to-height ratio.

### 3.2 Associations of IR indices with CVD

**Figure 1:**
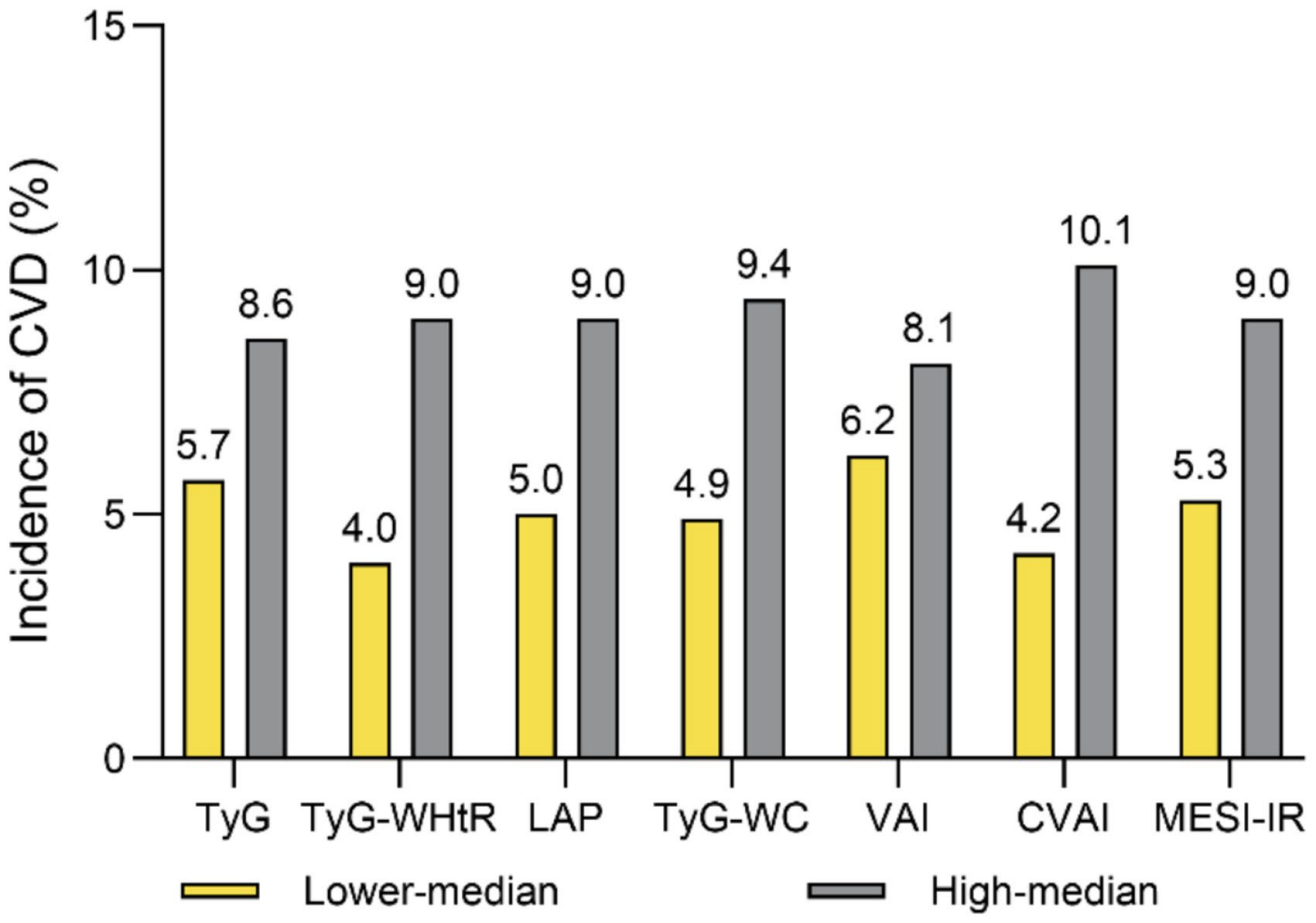
Incidence of CVD base on the median of seven IR indices in the total population. CVD cardiovascular disease, TyG triglyceride-glucose, VAI visceral adiposity index, CVAI Chinese visceral adiposity index, LAP lipid accumulation product, METS-IR metabolic score for insulin resistance, WC waist circumference, WHtR waist-to-height ratio.

**Figure 2:**
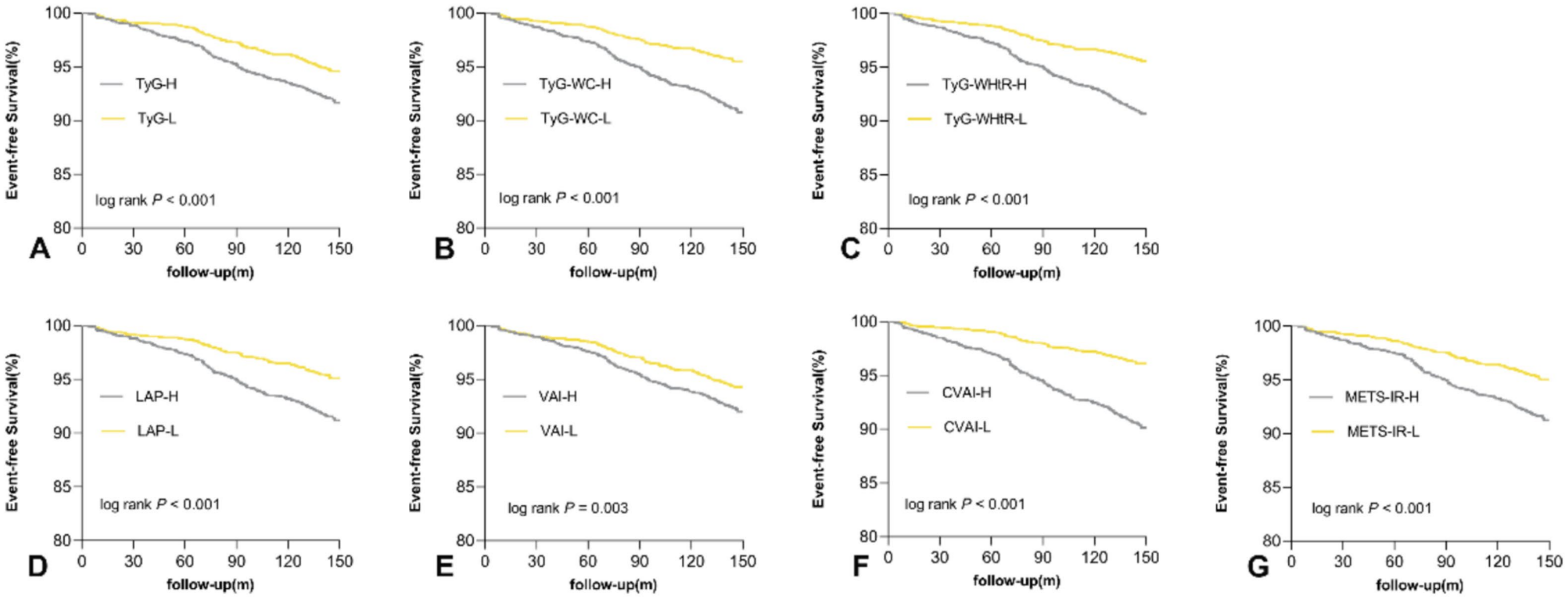
Kaplan–Meier curves for no CVD survival base on the median of TyG index **(A)**, TyG-WC **(B)**, TyG-WHtR **(C)**, LAP **(D)**, VAI **(E)**, CVAI **(F)**, METS-IR **(G)** in total population. CVD cardiovascular disease, TyG triglyceride-glucose, VAI visceral adiposity index, CVAI Chinese visceral adiposity index, LAP lipid accumulation product, METS-IR metabolic score for insulin resistance, WC waist circumference, WHtR waist-to-height ratio.

As shown in Figure 1, the incidence of CVD among individuals with a higher median of the TyG index (8.6% vs. 5.7%), TyG-WHtR (9.6% vs. 4.8%), LAP (9.0% vs. 5.4%), TyG-WC (9.4% vs. 4.9%), VAI (8.1% vs. 6.2%), CVAI (10.1% vs. 4.2%), and METS-IR (9.0% vs. 5.4%) was higher compared to a lower median (all *p* < 0.001). Moreover, Kaplan–Meier curve analysis for CVD revealed significant statistical differences between the higher- and lower-median groups (all log-rank *p* < 0.001; Figure 2).

The results of univariable Cox regression analyses revealed that individuals in high level of IR surrogates (TyG, TyG-WC, TyG-WHtR, LAP, VAI, CVAI and METS-IR) had increased risk of CVD(HR: 1.53, 95% CI:1.23–1.89, *p* < 0.001; HR: 2.01, 95% CI: 1.61–2.52, *p* < 0.001; HR: 2.16, 95% CI: 1.72–2.70, *p* < 0.001; HR: 3.62, 95% CI: 3.09–4.24, *p* = 0.001; HR: 1.36, 95% CI:1.10–1.68, *p* = 0.005; HR: 2.58, 95% CI: 2.00–3.18, *p* < 0.001; HR: 1.76, 95% CI: 1.42–2.19, *p* < 0.001; respectively). After adjusting confounders, this association still persisted (HR: 1.42, 95% CI:1.14–1.77, *p* = 0.002; HR: 1.67, 95% CI: 1.33–2.09, *p* < 0.001; HR: 1.79, 95% CI: 1.42–2.27, *p* < 0.001; HR: 1.67, 95% CI: 1.33–2.01, *p* < 0.001; HR: 1.41, 95% CI:1.13–1.76, *p* = 0.002; HR: 1.84, 95% CI: 1.45–2.35, *p* < 0.001; HR: 1.62, 95% CI: 1.30–2.02, *p* < 0.001; respectively). Compared to individuals with the lower median, CVAI was identified as the strongest risk marker for CVD, exhibiting the highest HR among other IR indices (unadjusted HR: 2.58, 95% CI: 2.00–3.18, *p* < 0.001; adjusted HR: 1.84, 95% CI: 1.45–2.35, *p* < 0.001; Table 2).

**Table 2:** Association of seven IR indices and the risk of CVD in total population.

Variable	HR (95% CI)			
	Unadjusted	*p*-value	Adjusted	*p*-value
TyG				
TyG-L	Ref		Ref	
TyG-H	1.53 (1.23–1.89)	<0.001	1.42 (1.14–1.77)	0.002
Per 1-unit increase	1.60(1.38–1.84)	0.001	1.57 (1.35–1.82)	<0.001
TyG-WC				
TyG-WC-L	Ref		Ref	
TyG-WC-H	2.01(1.61–2.52)	<0.001	1.67 (1.33–2.09)	<0.001
Per 1-unit increase	1.01 (1.01–1.01)	<0.001	1.01 (1.01–1.01)	<0.001
TyG-WHtR				
TyG-WHtR-L	Ref		Ref	
TyG-WHtR-H	2.16(1.72–2.70)	<0.001	1.79 (1.42–2.27)	<0.001
Per 1-unit increase	1.71(1.47–1.99)	<0.001	1.48 (1.26–1.76)	<0.001
LAP				
LAP-L	Ref		Ref	
LAP-H	3.62 (3.09–4.24)	<0.001	1.67 (1.33–2.01)	<0.001
Per 1-unit increase	1.02 (1.01–1.02)	0.001	1.01 (1.01–1.01)	<0.001
VAI				
VAI-L	Ref		Ref	
VAI-H	1.36(1.10–1.68)	0.005	1.41 (1.13–1.76)	0.002
Per 1-unit increase	1.03(1.01–1.05)	0.003	1.04 (1.02–1.06)	<0.001
CVAI				
CVAI-L	Ref		Ref	
CVAI-H	2.58(2.00–3.18)	<0.001	1.84 (1.45–2.35)	<0.001
Per 1-unit increase	1.01 (1.01–1.02)	<0.001	1.01 (1.01–1.01)	<0.001
METS-IR				
METS-IR-L	Ref		Ref	
METS-IR-H	1.76(1.42–2.19)	<0.001	1.62(1.30–2.02)	<0.001
Per 1-unit increase	1.05(1.04–1.07)	<0.001	1.05(1.03–1.07)	<0.001

HR, hazard ratio; CI, confidence interval; TyG triglyceride-glucose, VAI visceral adiposity index, CVAI Chinese visceral adiposity index, LAP lipid accumulation product, METS-IR metabolic score for insulin resistance, WC waist circumference, WHtR waist-to-height ratio. Adjusted model was adjusted for age, sex, education level, smoking, hypertension, diabetes, dyslipidemia, homocysteine, and creatinine.

**Figure 3:**
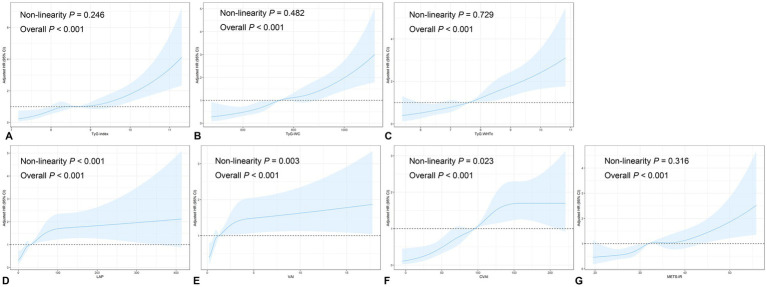
Restricted cubic spine curves for the association of TyG **(A)**, TyG-WC **(B)**, TyG-WHtR **(C)**, LAP **(D)**, VAI **(E)**, CVAI **(F)**, and METS-IR **(G)** with the risk of CVD in the adjusted model. TyG triglyceride-glucose, VAI visceral adiposity index, CVAI Chinese visceral adiposity index, LAP lipid accumulation product, METS-IR metabolic score for insulin resistance, WC waist circumference, WHtR waist-to-height ratio. Adjusted model was adjusted for age, sex, education level, smoking, hypertension, diabetes, dyslipidemia, homocysteine, and creatinine.

Given the association between IR indices and CVD, we further performed the nonlinear association of IR indices and the risk of CVD. The RCS curves were shown in Figure 3. In the multiple confounder correction model, linear association was detected between TyG index, TyG-WC, TyG-WHtR and METS-IR and CVD risk (all non-linearity *p* > 0.05; Figures 3A–C, G). However, LAP, VAI and CVAI demonstrates nonlinear relationship with CVD risk (all non-linearity *p* < 0.05; Figures 3D–F).

### 3.3 Diagnostic performance of IR indices for CVD

**Figure 4:**
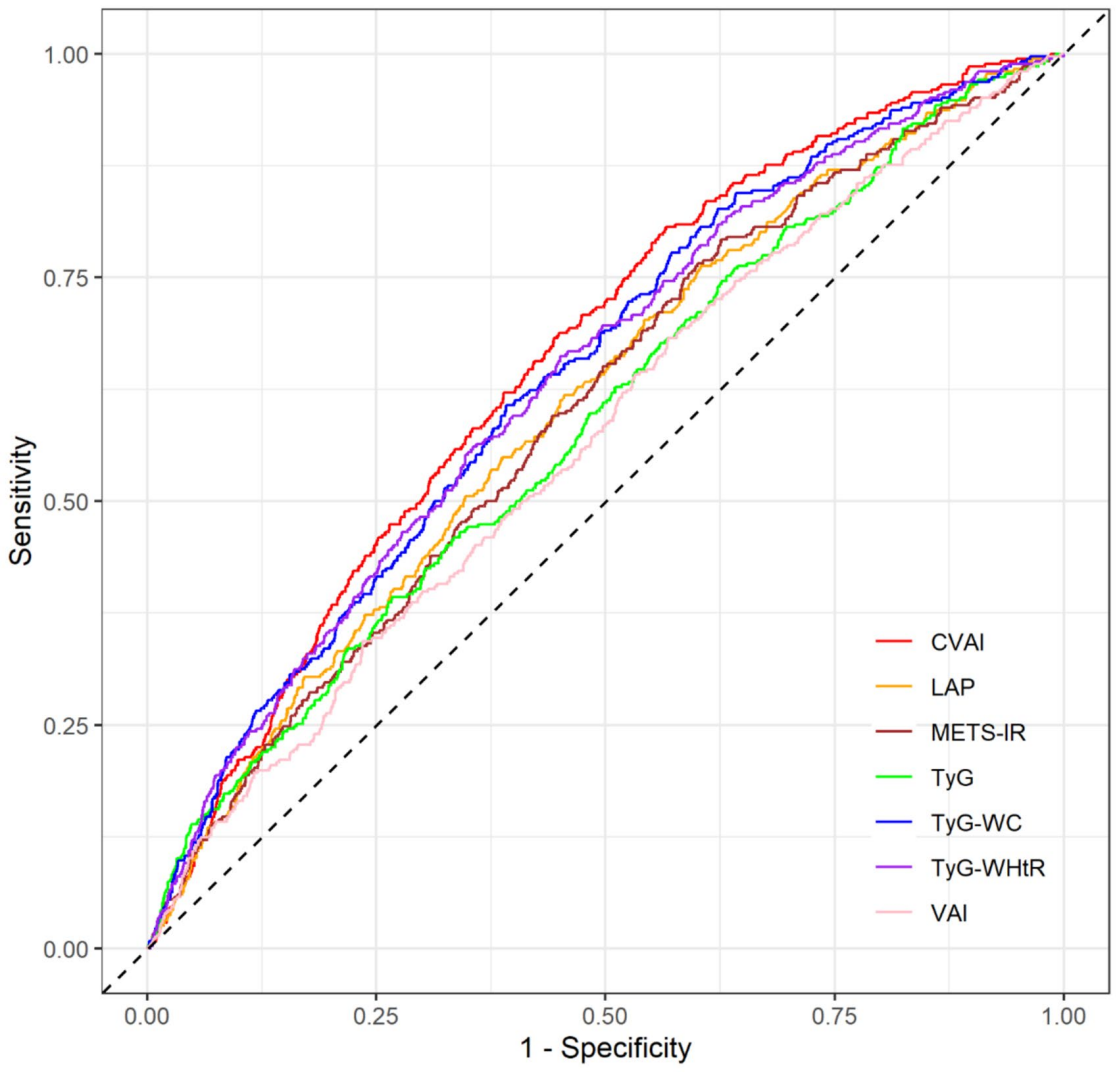
ROC curves evaluating the diagnostic performance of seven IR indices for CVD in the total population. TyG, Triglyceride-glucose; VAI, visceral adiposity index; CVAI, Chinese visceral adiposity index; LAP, lipid accumulation product; METS-IR, metabolic score for insulin resistance; WC, waist circumference; WHtR, waist-to-height ratio.

The ROC curve analysis was estimated and compared the diagnostic performance of each IR indices for CVD. CVAI had an AUC of 0.654 (95% CI 0.640–0.666), indicating modest discriminatory ability, which was the highest among all indices. In comparison, the TyG index had an AUC of 0.584 (95% CI: 0.571–0.598, little discriminatory ability), TyG-WC had an AUC of 0.636 (95% CI: 0.623–0.649, modest discriminatory ability), TyG-WHtR had an AUC of 0.613 (95% CI: 0.600–0.626, modest discriminatory ability), LAP had an AUC of 0.601 (95% CI: 0.588–0.614, modest discriminatory ability), VAI had an AUC of 0.563 (95% CI: 0.549–0.576, little discriminatory ability), and METS-IR had an AUC of 0.596 (95% CI: 0.583–0.610, little discriminatory ability). All between-group comparisons were statistically significant compared with CVAI (*P* for comparison < 0.001, except TyG-WC: *p* = 0.018; TyG-WHtR: *p* = 0.001; Supplementary Table 2; Figure 4). Moreover, the cut-off value, the sensitivity and specificity for each IR indices were calculated, respectively (Supplementary Table 2).

### 3.4 Incremental value of IR indices over original model for CVD

The addition of CVAI, compared to other IR indices including TyG index, TyG-WC, TyG-WHtR, LAP, VAI and METS-IR performed the maximum increased for CVD based on the based model (age, sex, smoking, education level, hypertension, dyslipidemia, diabetes, creatinine and homocysteine), with regard to NRI (0.358, 95% CI, 0.251–0.466; *p* < 0.001) and IDI (0.010, 95% CI, 0.006–0.013; *p* < 0.001). Adding other IR indices to the original model resulted in a significant but relatively minor enhancement effect (Table 3).

**Table 3:** Improvement in discrimination and risk reclassification for CVD risk after adding seven IR indices.

Characteristics	NRI		IDI	
	Estimate (95% CI)	*p*-value	Estimate (95% CI)	*p*-value
based model	Ref	–	Ref	–
based model +TyG	0.252(0.143–0.361)	<0.001	0.008 (0.004–0.012)	<0.001
based model +TyG-WC	0.305(0.196–0.414)	<0.001	0.011 (0.007–0.015)	<0.001
based model +TyG-WHtR	0.322(0.214–0.431)	<0.001	0.010 (0.006–0.014)	<0.001
based model +LAP	0.303(0.196–0.410)	<0.001	0.005 (0.011–0.008)	0.007
based model +VAI	0.266(0.160–0.372)	<0.001	0.002 (0.000–0.003)	0.015
based model +CVAI	0.358(0.251–0.466)	<0.001	0.010(0.006–0.013)	<0.001
based model +METS-IR	0.261(0.152–0.370)	<0.001	0.008(0.004–0.011)	<0.001

HR, hazard ratio; CI, confidence interval; TyG triglyceride-glucose, VAI visceral adiposity index, CVAI Chinese visceral adiposity index, LAP lipid accumulation product, METS-IR metabolic score for insulin resistance, WC waist circumference, WHtR waist-to-height ratio. Based model was adjusted for age, sex, education level, smoking, hypertension, diabetes, dyslipidemia, homocysteine, and creatinine.

### 3.5 Threshold effect and stratified analysis of CVAI on CVD

The two-piecewise Cox proportional hazards regression models were fitted the association of CVAI with the risk of CVD, and Table 4 showed that the threshold point for CVD was 96.21(*p* values for log-likelihood ratio < 0.001). After adjusting potential confounders, When the level of CVAI was below 96.21, CVAI was negatively associated with CVD risk (HR: 0.47 95% CI: 0.37–0.60; *p* < 0.001). When the level of CVAI exceeded the threshold point, the group of the CVAI was positively associated with CVD risk (HR: 2.11 95% CI: 1.66–2.67; *p* < 0.001; Table 4).

**Table 4:** Threshold effect analysis of CVAI on CVD.

CVD	Adjusted HR	
	(95% CI)	*p* for value
Total	1.88 (1.48–2.39)	<0.001
Fitting by two-piecewise Cox proportional risk model		
Inflection point	96.21	
CVAI < 96.21	0.47(0.37–0.60)	<0.001
CVAI ≥ 96.21	2.11(1.66–2.67)	<0.001
*p* for log-likelihood ratio	<0.001	

HR, hazard ratio, CI, confidence interval, CVAI, Chinese visceral adiposity index. Adjusted model was adjusted for age, sex, education level, smoking, hypertension, diabetes, dyslipidemia, homocysteine, and creatinine.

**Figure 5:**
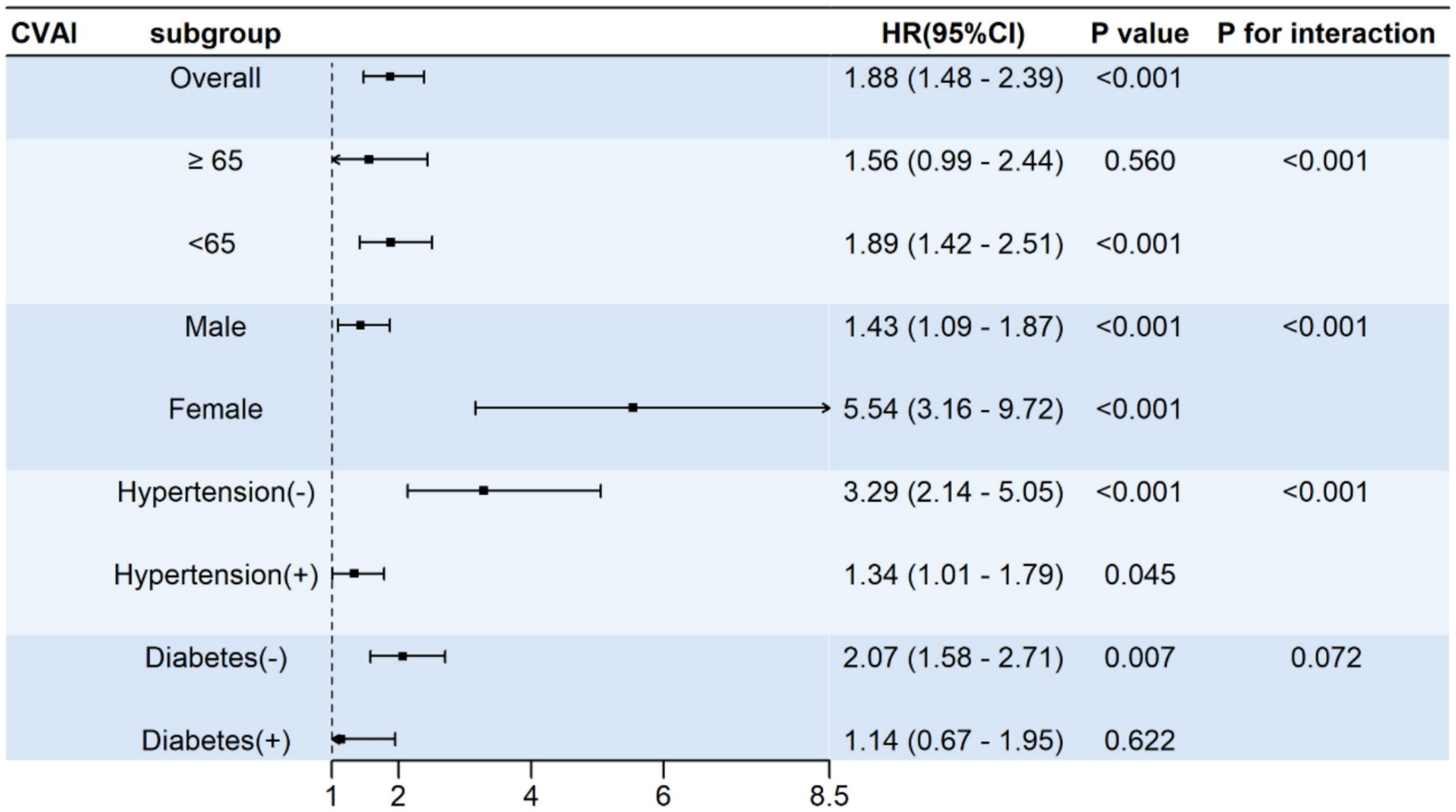
Stratified analyses of the associations between CVAI and CVD risk. HR, Hazard ratio; CI, confidence interval; CVAI, Chinese visceral adiposity index. Adjusted model was adjusted for age, sex, education level, smoking, hypertension, diabetes, dyslipidemia, homocysteine, and creatinine.

Compared to individuals with lower CVAI (< 96.21), those with higher CVAI (≥ 96.21) demonstrated a consistent CVD risk across various subgroups according to gender, hypertension and diabetes (Figure 5). There was an interaction between the CVAI and gender, age, or hypertension. Compared with lower CVAI, HR with a 95% CI for CVD was 1.89 (1.42–2.51) for participants aged less than 65 years, and 1.56 (0.99–2.44) for those aged 65 years and older. Compared to the hypertension and male group, stronger associations of higher CVAI with CVD risk was found in the subgroup with hon-hypertension and female.

### 3.6 Sensitivity analyses

Since each IR surrogate was affected by anti-diabetes and anti-dyslipidemia treatments, sensitivity analyses excluding participants with these conditions were conducted. These analyses assessed the association between CVD risk and seven IR indices (TyG, TyG-WC, TyG-WHtR, LAP, VAI, CVAI, and METS-IR) and confirmed similar results (Supplementary Tables 3, 4). Excluding events occurring in the early 2 years of follow-up, the results were highly consistent with our primary findings, confirming the stability and generalizability of our conclusions (Supplementary Table 5).

## 4 Discussion

This prospectively cohort study assessed and compared the predictive value of seven IR indices for CVD. The major findings of this study are as follows: First, different IR indices including TyG, TyG-WC, TyG-WHtR, LAP, VAI, CVAI, and METS-IR were independently and positively associated with the risk of CVD. Second, CVAI, compared to TyG, TyG-WC, TyG-WHtR, LAP, VAI, and METS-IR, had the highest risk for CVD and greatest power for predicting and stratifying the risk of CVD. Third, there was a non-linear association between the CVAI and the risk of CVD and the threshold point was 96.21. Finally, Stratified analysis suggested that the association of CVAI (higher CVAI group: ≥ 96.21; lower CVAI group: < 96.12) with CVD was modified by age (≥ 65 years and < 65 years), sex, and hypertension.

Insulin resistance, first reported by Harold in 1936 (17), is known to contribute to hyperinsulinemia and hyperglycemia and is considered a central aspect of a range of cardiometabolic abnormalities, including diabetes, hypertension, obesity, and dyslipidemia, which in turn leads to CVD and may affect prognosis (18, 19). The HIEG clamp technique, the gold standard for evaluating IR, is expensive and complex, which only is suitable for small-scale testing of insulin (20). HOMA-IR is calculated using FBG and fasting insulin and is suggested to be associated with CVD (21). However, HOMA-IR is not suitable for individuals with applying exogenous insulin and the measurement of fasting insulin is not routinely conducted in clinical practice, which makes HOMA-IR unsuitable for widespread clinical application. Thus, to assess insulin resistance more conveniently, many alternative indices have been developed, including the TyG index, TyG-WC, TyG-WHtR, LAP, VAI, CVAI, and METS-IR.

Numerous studies have revealed that IR indices are strongly associated with the risk of CVD. A meta-analysis including 8 cohort studies with 5,731,294 participants suggested that elevated TyG index increased CVD risk by 61%. Similarly, the TyG index, as continuous variable, was positively associated with the risk of CVD (22). Jaramillo et al., based on the PURE study, which registered 141,243 participants from 22 countries in 5 continents, demonstrated that the TyG index was strongly associated with MI, stroke, and cardiac mortality (23). Current TyG-related parameters, including TyG-BMI, TyG-WC, and TyG-WHtR, have been proven to be independently related to the risk of CVD (24–26). Moreover, Xia et al. compared the predictive value of the TyG index and its related parameters for ASCVD and found that TyG-WC and TyG-WHtR posed a higher risk of ASCVD than the TyG index (27). A prospective study revealed that CVAI had the best ability to predict cardiovascular events among diabetes individuals compared with VAI and LAP (28). Currently, METS-IR, as reported by Bello-Chavolla (29), was related to coronary artery calcification (30) and to increase the risk of CVD by 80% among populations without history of CVD (31). Findings from Pan et al. further showed that, compared to other IR indices (TyG index, TyG-BMI, TyG-WC, TyG-WHtR, and HOMA-IR), METS-IR had the highest diagnostic value of major adverse cardiovascular events with an AUC of 0.635 in patients with diabetes (32). Given that individuals with diabetes exhibit elevated levels of IR compared to the general population, findings from studies conducted with the diabetic population may not be directly inferable to the broader population. Liu et al. included 6,393 individuals from eastern of China suggested the CVAI had the highest associated with the risk of coronary heart disease (CHD) compared to TyG, TyG-BMI, TyG-WC, TyG-WHtR, VAI, and LAP. However, it solely focused on the association of different IR indices with the risk of CHD, and it remains unclear whether CVAI or METS-IR demonstrates superior predictive ability for CVD. Most previous studies were based on special diseases to explored the efficacy of some IR indices for predicting CVD or MACE. There is little research comparing the value of various IR indices on prediction for CVD among general population without history of CVD.

To fill this gap, this present study investigated the association of various IR indices on the risk of CVD and found that CVAI had the highest predictive power than other IR indices on the prediction and stratification for CVD. The study included 5,194 individuals without history of CVD. Over a follow-up of 12 years, 373 individuals had CVD. Consisted with previous study, individuals with higher levels of various IR markers exhibited a higher risk for CVD than those with lower levels of IR markers. These findings suggested that various of IR indices are independent risk factors for CVD. Notably, compared to other IR indices, CVAI not only demonstrated superior predictive value for the risk of CVD among individuals without history of CVD but also provided the greatest incremental risk stratification when added to original model. A potential explanation for this phenomenon, compared to the TyG index and its related indices, is that CVAI includes the HDL-C, which is a protective biomarker of CVD with antiatherosclerotic effects (33). Moreover, while VAI and LAP were originally designed to assess adipose tissue in Caucasians (8), they may not be applicable for evaluating the risk of CVD among Chinese population as effectively as CVAI. Compared to METS-IR, CVAI also incorporates the age factor, which is recognized as an independent cardiovascular-related factor. Previous studies have demonstrated that CVAI is associated with DM, hypertension, and carotid plaque. In addition, a cohort study showed that elevated CVAI is linked to an increased risk of stroke (34). These usually have similar or common risk factors with CVD. Abdominal obesity is closely associated with insulin resistance, which increases chronic inflammation, reduces adiponectin secretion, and enhances the secretion of oxidized low-density lipoprotein (35, 36). These factors ultimately contribute to the development of atherosclerosis.

We further performed the non-linear association of CVAI and the risk of CVD by the RCS curve. There was a non-linear association between CVAI and CVD risk, and threshold analysis showed a turning point at 96.21. When the level of CVAI exceeded 96.21, each unit increase in CVAI increased a 2.11-fold the risk of CVD. Conversely, when CVAI was below 96.21, the risk of CVD was negatively associated with level of CVAI. This phenomenon may relate to the J-shape association of HDL-C and CVD risk (37). The RCS curve indicated that the level of CVAI above 96.21 in individuals without the history of CVD was associated with increased risk of CVD, suggesting that this point is a valuable reference and could be recognized as an early warning sign of lifestyle changes among general population and to remind physicians to take early intervention to decrease the prevalence of CVD. Further large-scale and multicenter studies should be conduct to evaluate the optimal threshold CVAI level. The association between CVAI and CVD risk was more prominent among individuals aged<65 years, female, and non-hypertension. Furthermore, the association of elevated CVAI (**≥**96.21) with the risk of CVD was stronger among individuals without hypertension or among female. It could be supported by Zhang et al. that non-hypertensive participants with higher level of CVAI tend to have a higher risk of stroke (34). This identified threshold of 96.21 for CVAI may serve as a simple and practical tool for population-level screening of cardiovascular disease risk in clinical and community settings. In addition, CVAI could potentially be used in combination with traditional risk prediction models such as the Pooled Cohort Equations to further improve risk stratification. Notably, individuals with a CVAI level greater than 96.21 had an increased risk for CVD among those aged < 65 years and there was a significant interaction between the age and incident CVD. However, in the elderly population, this association may not persist. A possible explanation for this observation is that individuals over the age of 65 tend to have a higher prevalence of ectopic fat deposits, which may obscure the effect of CVAI (38). Additionally, participants aged < 65 years have a longer duration of exposure to excess visceral fat compared with those aged > 65 years. What’s more, a study included the Shanghai Suburban Adult Cohort and Biobank (SSACB) also found that CVAI’s predictive ability for CVD risk is more prominent among women (39). However, the correlation between CVAI and carotid plaque risk maybe lower in females than in males (40).

IR contributes to hyperinsulinemia, which leads to hyperglycemia, oxidative stress, and inflammatory reaction (18). In addition, IR is closely associated with endothelial dysfunction, vulnerable plaques, and visceral obesity (41, 42), all of which are recognized as contributing to the pathogenesis for atherosclerosis. Nitric oxide, a vasodilator and anti-atherogenic sclerosing factor (43, 44), is deficient in populations with IR, leading to accelerated atherosclerosis (44). Moreover, IR has also been shown to be related to hypertension (45), a prothrombotic state (46), atrial fibrillation, and vascular smooth muscle cell proliferation, which may explain the predictive value of IR indices for CVD. What’s more, the superior performance of CVAI for predicting CVD risk may be explained by its unique combination of components that collectively capture visceral adiposity-related metabolic dysfunction, which is particularly prominent in the Chinese population. First, waist circumference (WC) and BMI directly reflect central and overall adiposity, a key driver of insulin resistance and chronic low-grade inflammation (47, 48). Second, triglycerides (TG) and HDL-C characterize the atherogenic lipid profile commonly associated with visceral fat accumulation (49). Third, age reflects the cumulative effect of long-term metabolic stress and regulates physiological and pathological processes through multiple metabolic pathways, such as redox balance, endoplasmic reticulum stress, mitochondrial function, and nutrient-sensing networks (50). So CVAI can more precisely identify individuals in the Chinese population who are at high risk of CVD by combining these important risk variables. Lastly, several statistical comparisons were performed across different insulin resistance indices without formal adjustment for multiple comparisons, which may increase the risk of type I error. This should be taken into account when interpreting the present results.

To the best of our knowledge, the present study is the first population-based prospective cohort study to compare the performance of seven insulin resistance surrogates for CVD prediction and risk stratification among CVD-free Chinese adults over a 12-year follow-up period. All of results provided new evidence for the primary prevention of CVD by early recognizing population with CVAI **≥** 96.21. However, this study has several limitations. First, HOMA-IR data were unavailable in this study, precluding direct comparisons between these seven IR indices and this reference measure. Second, the participants included in this study were all from the northern part of China. Thus, the generalizability of these findings to broader populations is limited. The research results need to be further verified in multiple centers and among different ethnic groups. In addition, there may be potential confounders that could impact the risk of CVD in spite of multivariate adjustment and stratification analysis. Several potential confounding factors, including physical activity, dietary habits, family history of cardiovascular disease, and detailed medication use, were not available in the present study. Moreover, this study only evaluates the association IR indices with the risk of CVD by baseline, and the association with IR indices variability and CVD risk remains unknown, which further needs multicenter prospective studies. This study has an additional limitation. The research was conducted based on a routine health screening cohort, in which fasting insulin measurement is not included in the standard laboratory panel. Consequently, fasting insulin data were unavailable for all participants, making it impossible for us to calculate the HOMA-IR index and further compare it with other indicators of IR. Finally, the integration of novel biomarkers with traditional cardiovascular risk factors shows great potential in improving risk stratification (51). In the future, combining these indicators with machine learning algorithms may further enhance the accuracy and efficiency of risk prediction for high-risk populations, thereby facilitating early identification and targeted intervention of individuals at elevated CVD risk.

## 5 Conclusion

Overall, this prospective study revealed that all IR indices were significantly associated with the risk of CVD among population without history of CVD. Compared to other IR indices, CVAI is most strongly associated with the risk of CVD and provides the greatest additional predictive value for CVD, especially for women under 65 years. These findings showed that adding CVAI into risk algorithms in general population can improve risk stratification.

## Data Availability

The raw data supporting the conclusions of this article will be made available by the authors, without undue reservation.
