# Supplementary material for: Chinese visceral adiposity index outperforms insulin resistance indices in predicting cardiovascular disease risk: a 12-year prospective cohort study
Source: Front Nutr. 2026 May 5;13:1795750. doi: 10.3389/fnut.2026.1795750 (PMC13183553; doi:10.3389/fnut.2026.1795750)
Supplement: Supplementary file 1 [file Data_Sheet_1.pdf]

Supplemental files

Supplemental Table 1. The formulas for calculation of various IR indices.

IR indices	Formulas
TyG index	ln (TG (mg/dl) ×FPG (mg/dl)/2)
TyG-WC	WC* ln (TG (mg/dl) ×FPG (mg/dl)/2)
TyG-WHtR	WHtR * ln (TG (mg/dl) ×FPG (mg/dl)/2)
VAI	Male: [WC (cm)/ (39.68 + 1.88 × BMI)] × [fasting TG (mmol/L)/1.03] × [1.31/fasting HDL- C (mmol/L)]
	Female: [WC (cm)/ (36.58 + 1.89 × BMI)] × [fasting TG (mmol/L)/0.81] × [1.52/fasting HDL- C (mmol/L)]
CVAI	Male: − 267.93 + 0.68 × age + 0.03 × BMI + 4.00 × WC (cm) + 22.00 × Lg [fasting TG (mmol/L)] − 16.32 × fasting HDL- C (mmol/L)
	Female: − 187.32 + 1.71 × age + 4.32 × BMI + 1.12 × WC (cm) + 39.76 × Lg [fasting TG (mmol/L)] − 11.66 × fasting HDL- C (mmol/L)
LAP	Male: [WC (cm) − 65] × [fasting TG (mmol/L)]
	Female: [WC (cm) − 58] × [fasting TG (mmol/L)]
METS-IR	Ln (2*FBG + TG) * BMI (Ln (HDL - C)

TyG triglyceride- glucose, VAI visceral adiposity index, CVAI Chinese visceral adiposity index, LAP lipid accumulation product, METS-IR metabolic score for insulin resistance, WC waist circumference, WHtR waist-to-height ratio, BMI body mass body, HDL-C high-density lipoprotein-cholesterol, FPG fasting plasma glucose, TG triglyceride.

Supplemental Table 2. Predictive performance of seven IR indices for CVD risk.

Variable	AUC			Cut-off	Sensitivity (%)	Specificity (%)
	Est.(95%CI)	*P* value	*P* for comparison			
CVAI	0.654(0.640-0.666)	<0.001	Ref	86.11	80.2	43.5
TyG	0.584(0.571-0.598)	<0.001	<0.001	8.43	74.6	37.3
TyG-WC	0.636(0.623-0.649)	<0.001	0.018	771.81	59.2	60.9
TyG-WHtR	0.613(0.600-0.626)	<0.001	<0.001	4.61	59.2	59.5
METS-IR	0.596(0.583-0.610)	<0.001	<0.001	29.97	78.0	37.4
LAP	0.601(0.588-0.614)	<0.001	<0.001	32.89	60.6	54.6
VAI	0.563(0.549-0.576)	<0.001	<0.001	1.11	67.3	43.3

AUC area under the curve, CI confidence interval, TyG triglyceride- glucose, VAI visceral adiposity index, CVAI Chinese visceral adiposity index, LAP lipid accumulation product, METS-IR metabolic score for insulin resistance, WC waist circumference, WHtR waist-to-height ratio.

Supplemental Table 3. Association of seven IR indices and the risk of CVD in individuals excluding anti-dyslipidemia.

Variable	HR (95%CI)			
	Unadjusted	*P*-value	Adjusted	*P*-value
TyG				
TyG-L	Ref		Ref	
TyG-H	1.53 (1.24 – 1.91)	<0.001	1.43 (1.15 - 1.79)	0.001
Per 1-unit increase	1.59 (1.37 -1.84)	<0.001	1.56 (1.33 - 1.81)	<0.001
TyG-WC				
TyG-WC-L	Ref		Ref	
TyG-WC-H	2.02 (1.61 – 2.53)	<0.001	1.66 (1.32 – 2.10)	<0.001
Per 1-unit increase	1.00 (1.00 -1.01)	<0.001	1.00 (1.00 - 1.01)	<0.001
TyG-WHtR				
TyG-WHtR-L	Ref		Ref	
TyG-WHtR-H	2.13(1.70 – 2.67)	<0.001	1.77 (1.40 – 2.25)	<0.001
Per 1-unit increase	1.71 (1.46 – 1.99)	<0.001	1.48 (1.25 – 1.76)	<0.001
LAP				
LAP-L	Ref		Ref	
LAP-H	1.78 (1.42 – 2.22)	<0.001	1.67(1.33 – 2.09)	<0.001
Per 1-unit increase	1.00 (1.00 - 1.01)	<0.001	1.00 (1.00 - 1.01)	<0.001
VAI				
VAI-L	Ref		Ref	
VAI-H	1.38 (1.11 – 1.71)	<0.001	1.44 (1.15 – 1.80)	0.001
Per 1-unit increase	1.03 (1.01 - 1.05)	<0.001	1.04 (1.02 - 1.06)	<0.001
CVAI				
CVAI-L	Ref		Ref	
CVAI-H	2.55 (2.02 – 3.23)	<0.001	1.86 (1.45 – 2.37)	<0.001
Per 1-unit increase	1.01 (1.01 - 1.02)	<0.001	1.01 (1.01 - 1.01)	<0.001
METS-IR				
METS-IR -L	Ref		Ref	
METS-IR -IR -H	1.81 (1.45 – 2.26)	<0.001	1.66 (1.33 – 2.08)	<0.001
Per 1-unit increase	1.06 (1.04 - 1.07)	<0.001	1.05 (1.03 - 1.06)	<0.001

HR, hazard ratio; CI, confidence interval; TyG triglyceride- glucose, VAI visceral adiposity index, CVAI Chinese visceral adiposity index, LAP lipid accumulation product, METS-IR metabolic score for insulin resistance, WC waist circumference, WHtR waist-to-height ratio

Adjusted model was adjusted for age, sex, education level, smoking, hypertension, diabetes, dyslipidemia, homocysteine, creatinine.

Supplemental Table 4. Association of seven IR indices and the risk of CVD in individuals excluding anti-diabetes.

Variable	HR (95%CI)			
	Unadjusted	*P*-value	Adjusted	*P*-value
TyG				
TyG-L	Ref		Ref	
TyG-H	1.65 (1.31 - 2.08)	<0.001	1.59 (1.26 – 2.01)	<0.001
Per 1-unit increase	1.68 (1.44 -1.96)	<0.001	1.70 (1.45 – 2.00)	<0.001
TyG-WC				
TyG-WC-L	Ref		Ref	
TyG-WC-H	2.16 (1.69 – 2.74)	<0.001	1.79 (1.41- 2.29)	<0.001
Per 1-unit increase	1.00 (1.00 -1.01)	<0.001	1.01 (1.01 - 1.01)	<0.001
TyG-WHtR				
TyG-WHtR-L	Ref		Ref	
TyG-WHtR-H	2.01(1.62 – 2.60)	<0.001	1.76 (1.38 – 2.26)	<0.001
Per 1-unit increase	1.69 (1.44 – 2.01)	<0.001	1.52 (1.26 – 1.82)	<0.001
LAP				
LAP-L	Ref		Ref	
LAP-H	1.83(1.45 – 2.31)	<0.001	1.76 (1.38 – 2.24)	<0.001
Per 1-unit increase	1.00(1.00 - 1.01)	<0.001	1.01 (1.00 - 1.01)	<0.001
VAI				
VAI-L	Ref		Ref	
VAI-H	1.34 (1.06 - 1.67)	0.013	1.44 (1.14 – 1.82)	0.003
Per 1-unit increase	1.03(1.01-1.05)	0.003	1.04 (1.02 - 1.07)	<0.001
CVAI				
CVAI-L	Ref		Ref	
CVAI-H	2.85 (2.21 – 3.68)	<0.001	2.09 (1.60 – 2.72)	<0.001
Per 1-unit increase	1.01 (1.01 - 1.02)	<0.001	1.01 (1.01 - 1.01)	<0.001
METS-IR				
METS-IR -L	Ref		Ref	
METS-IR -H	1.88 (1.48 – 2.37)	<0.001	1.74 (1.37 – 2.21)	<0.001
Per 1-unit increase	1.06 (1.04 - 1.08)	<0.001	1.06 (1.04 - 1.08)	<0.001

HR, hazard ratio; CI, confidence interval; TyG triglyceride-glucose, VAI visceral adiposity index, CVAI Chinese visceral adiposity index, LAP lipid accumulation product, METS-IR metabolic score for insulin resistance, WC waist circumference, WHtR waist-to-height ratio

Adjusted model was adjusted for age, sex, education level, smoking, hypertension, diabetes, dyslipidemia, homocysteine, creatine.

Supplemental Table 5. Association of seven IR indices and the risk of CVD in individuals excluding early 2-year CVD events.

Variable	HR (95%CI)			
	Unadjusted	*P*-value	Adjusted	*P*-value
TyG				
TyG-L	Ref		Ref	
TyG-H	1.48 (1.18 – 1.86)	<0.001	1.38 (1.10 - 1.74)	0.001
Per 1-unit increase	1.55 (1.32 - 1.81)	<0.001	1.52 (1.29 - 1.78)	<0.001
TyG-WC				
TyG-WC-L	Ref		Ref	
TyG-WC-H	1.95 (1.54 – 2.47)	<0.001	1.60 (1.26 – 2.03)	<0.001
Per 1-unit increase	1.00 (1.00 - 1.01)	<0.001	1.00 (1.00 - 1.01)	<0.001
TyG-WHtR				
TyG-WHtR-L	Ref		Ref	
TyG-WHtR-H	2.05 (1.62 – 2.59)	<0.001	1.70 (1.33 – 2.17)	<0.001
Per 1-unit increase	1.65 (1.40 – 1.94)	<0.001	1.42 (1.19 – 1.69)	<0.001
LAP				
LAP-L	Ref		Ref	
LAP-H	1.72 (1.36 – 2.17)	<0.001	1.61 (1.27 – 2.04)	<0.001
Per 1-unit increase	1.00 (1.00 - 1.01)	<0.001	1.00 (1.00 - 1.01)	<0.001
VAI				
VAI-L	Ref		Ref	
VAI-H	1.35 (1.08 – 1.69)	0.001	1.40 (1.11 – 1.76)	0.001
Per 1-unit increase	1.03 (1.01 - 1.05)	<0.001	1.04 (1.02 - 1.06)	<0.001
CVAI				
CVAI-L	Ref		Ref	
CVAI-H	2.45 (1.92 – 3.12)	<0.001	1.79 (1.39 – 2.30)	<0.001
Per 1-unit increase	1.01 (1.01 - 1.02)	<0.001	1.01 (1.01 - 1.01)	<0.001
METS-IR				
METS-IR-L	Ref		Ref	
METS-IR-H	1.75 (1.39 – 2.20)	<0.001	1.60 (1.27 – 2.02)	<0.001
Per 1-unit increase	1.05 (1.04 - 1.07)	<0.001	1.04 (1.03 - 1.06)	<0.001

HR, hazard ratio; CI, confidence interval; TyG triglyceride- glucose, VAI visceral adiposity index,

CVAI Chinese visceral adiposity index, LAP lipid accumulation product, METS-IR metabolic score for insulin resistance, WC waist circumference, WHtR waist-to-height ratio

Adjusted model was adjusted for age, sex, education level, smoking, hypertension, diabetes, dyslipidemia, homocysteine, creatine.

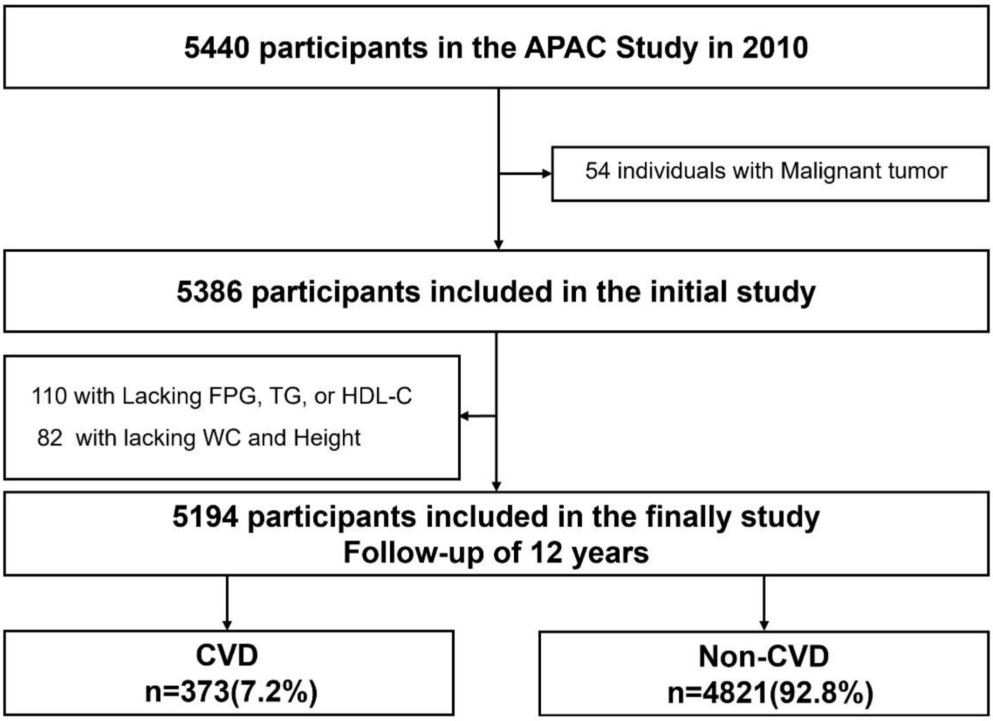

Supplemental Figure 1. Flow chart of this study. APAC Asymptomatic Polyvascular Abnormalities Community. CVD cardiovascular disease, HDL-C high-density lipoprotein-cholesterol, FPG fasting plasma glucose, TG triglyceride, WC waist circumference.

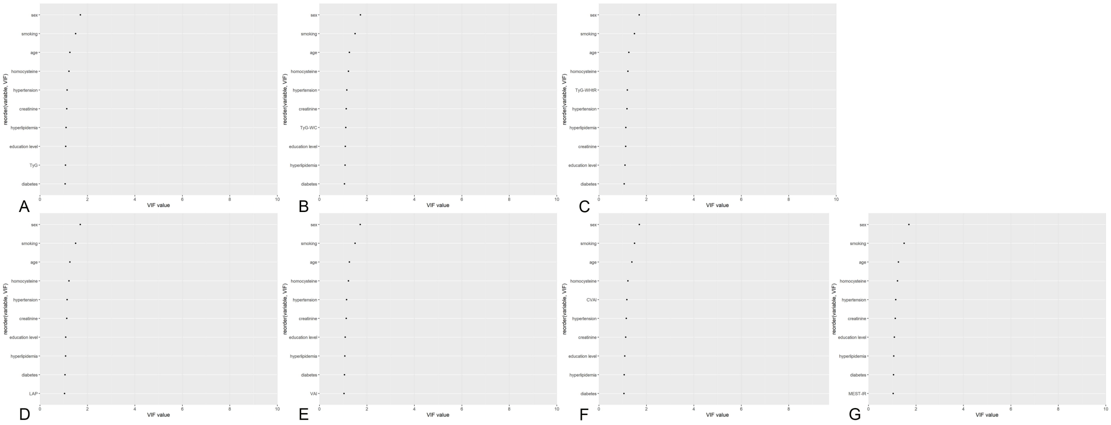

Supplemental Figure 2. The variance inflation factor (VIF) values for all variables in our adjusted model.

TyG triglyceride-glucose, VAI visceral adiposity index, CVAI Chinese visceral adiposity index, LAP lipid accumulation product, METS-IR metabolic score for insulin resistance, WC waist circumference, WHtR waist-to-height ratio

Adjusted model was adjusted for age, sex, education level, smoking, hypertension, diabetes, dyslipidemia, homocysteine, creatine.
